# 4288 Identifying Predictive Variables of High-Intensity Binge Drinking Through the Use of a Machine Learning Algorithm

**DOI:** 10.1017/cts.2020.399

**Published:** 2020-07-29

**Authors:** James Keoni Morris, Josh L. Gowin, Melanie L. Schwandt, Nancy Diazgranados, Vijay A. Ramchandani

**Affiliations:** 1 National Institutes of Health; 2 University of Colorado Denver; 3 National Insititute on Alcohol Abuse and Alcoholism; 4 National Institute on Alcohol Abuse and Alcoholism

## Abstract

OBJECTIVES/GOALS: To test if a machine learning algorithm could predict a person’s capacity to binge drink and explore what measures might be important for identifying individuals at risk for high-intensity binge drinking behaviors. METHODS/STUDY POPULATION: The sample included 1177 (474 female) non-treatment-seeking drinkers (age: 18-91 years), that were assigned to a group based on their heaviest drinking day reported in a 90-Day Alcohol Timeline Followback questionnaire. The groups were Non-Bingers (female: 12 drinks, male:>15 drinks). The sample was divided into a training sample (N = 884) and a testing sample (N = 293). A machine learning algorithm called random forest was then used to generate a predictive model based on measures of substance use, personality traits, and trauma. The model was applied to the testing sample to determine accuracy. RESULTS/ANTICIPATED RESULTS: The first model correctly assigned 190 out of 293 subjects, giving it a total error rate of 0.35, with lowest rates for non-binge (0.19) and high-intensity (0.18), while medium-intensity had the highest error rate (0.86). The most important variables for the accuracy of the model included: total score on the Alcohol Use Disorder Identification Test, first five sub-score of the Self-Reported Effects of Alcohol, Compulsive Drinking subscale, and presence of a current psychiatric diagnosis. As a follow-up analysis, we built and tested another random forest model without the use of drinking dependence measures. This model had a total error rate of 0.39, and introduced other important variables such as smoking behaviors, perceived stress, IQ, and number of negative life events. DISCUSSION/SIGNIFICANCE OF IMPACT: Our study showed that it was possible for a machine learning algorithm to predict binge drinking intensity better than chance. Drinking patterns were the most robust predictors, and stress, IQ, and psychiatric diagnoses were also useful in predicting binge drinking intensity.

